# Draft Genome Sequence of “*Candidatus* Methanomethylophilus” sp. 1R26, Enriched from Bovine Rumen, a Methanogenic Archaeon Belonging to the *Methanomassiliicoccales* Order

**DOI:** 10.1128/genomeA.01734-15

**Published:** 2016-02-18

**Authors:** Samantha Joan Noel, Ole Højberg, Tim Urich, Morten Poulsen

**Affiliations:** a Department of Animal Science, Aarhus University, Tjele, Denmark; b Department of Ecogenomics and Systems Biology, University of Vienna, Vienna, Austria; c Institute of Microbiology, Ernst-Moritz-Arndt University Greifswald, Greifswald, Germany; d Danish Technological Institute, Aarhus, Denmark

## Abstract

Here, we present the draft genome of “*Candidatus* Methanomethylophilus” sp. 1R26, a member of the newly described *Methanomassiliicoccales* order of *Euryarcheaota*. The enrichment culture was established from bovine rumen contents and produced methane from trimethylamine and methanol. The draft genome contains genes for methanogenesis from methylated compounds.

## GENOME ANNOUNCEMENT

Fermentation in the rumen by microbial consortia allows ruminant animals to utilize nondigestible cellulolistic feed, however, with methane as an end product. Methane lost from the animal is a contributor to global warming and represents a loss of energy from the animal. Methanogens from the order *Methanomassiliicoccales* (previously called Rumen Cluster C, RCC [1], or *Methanoplasmatales* [2]) represent a large group of methane producing archaea present in the rumen (3). Until now, this order contained only seven published genomes and one isolate in pure culture (4–9). Despite advances in culturing techniques, no pure cultures from the rumen environment exist.

Cultures were enriched from the rumen content of a Danish dairy cow using RM02 media (10) supplemented with sodium acetate, sodium formate, trimethylamine, and methanol. Enrichment culture 1R26 produced methane and contained *Methanomassiliicoccales*-associated 16S rRNA genes enriched to 60% of the total prokaryotic 16S rRNA genes as detected by qPCR, using previously described primers (11). DNA was extracted with a ZR-fungal/bacterial DNA kit (Zymo Research). A 454 pyrotag survey of the enrichment culture with universal prokaryotic primers 515F and R806, covering the V4-5 region of the 16S rRNA gene (12), revealed the presence of one single archaeal operational taxonomic unit (OTU) that was classified as belonging to *Methanomassiliicoccales*. The enrichment culture was sequenced on an Ion Torrent PGM sequencer (318 chip, Life Technologies) resulting in 6,389,036 reads. The reads were quality trimmed with Trimmomatic (13) and assembled into contigs using SPAdes v3.6.1 (14). *Methanomassiliicoccales*-associated contigs were binned on the basis of G+C content and coverage, and were annotated with Prokka (15). Binned contigs had greater than 200× coverage with a G+C range between 45 and 64%. Genome completeness of 92.98% with a contamination of 0.8% was estimated with CheckM (16).

“*Ca.* Methanomethylophilus” sp. 1R26 has a draft genome of 1,723,106 bp in 50 contigs, with a G+C content of 60.4% and encodes 2,076 genes, 37 tRNAs, and 5 rRNA genes (1 16S, 1 23S, and 3 5S). One clusters of regularly interspaced short palindromic repeat (CRISPR) loci and its associated *cas* gene was identified using CRISPRfinder (17). The 16S and 23S rRNA genes were found on the same contig separated by 21,855 bases and the 5S rRNA genes were found throughout the genome on separate contigs. The 1R26 16s rRNA gene is 87% similar to that of *Methanomassiliicoccus luminyensis* B10, the type species and only isolate of this order. The closest genome was “*Ca.* Methanomethylophilus alvus” sp. Mx1201 with 98% 16S rRNA similarity, however, whole-genome comparisons using digital DNA-DNA hybridization, with 20.2% ± 2.31% identity (http://ggdc.dsmz.de/distcalc2.php) and average nucleotide identity of 79.48% (http://enve-omics.ce.gatech.edu/), verify that these are not the same species. Members of the *Methanomassiliicoccales* order are proposed to be hydrogen-dependent methylotrophs. In support of this, genes for the utilization of mono-, di-, and trimethylamine and methanol for methanogenesis from methylated compounds were found, whereas genes specific to the hydrogenotrophic pathway of methanogenesis were absent.

### Nucleotide sequence accession numbers.

This whole-genome shotgun project has been deposited at DDBJ/EMBL/GenBank under the accession no. LOPS00000000. The version described in this paper is version LOPS01000000.
